# Dental Implants Placed in Fresh Human Extraction Sockets Without Osteotomy: A Case Series

**DOI:** 10.1002/cre2.70159

**Published:** 2025-06-11

**Authors:** Thanos Dounis, Valeria Jimenez Ortiz

**Affiliations:** ^1^ Private Practice Limited to Periodontics and Dental Implants Fredericksburg Virginia USA; ^2^ Harvard School of Dental Medicine Boston Massachusetts USA

**Keywords:** bone remodeling, case reports, dental implants, osseointegration

## Abstract

**Objectives:**

This case series evaluates healing parameters of immediately placed implants in fresh extraction sockets without osteotomies. It examines resonance frequency analysis (RFA) trends and radiographic bone changes from placement to prosthetic restoration.

**Material and Methods:**

The study reports on eight patients with 10 implants using a self‐tapping, knife‐shaped implant with progressive thread design and anatomical healing abutments. RFA values and standardized radiographs were recorded at three time points from placement to prosthetic restoration.

**Results:**

Implants were placed in molar, premolar, and incisor sockets. All implants achieved insertion torque greater than 35 Ncm and high ISQ values. The median reported bone remodeling was 0.35 mm from implant placement to definitive restoration. RFA revealed a median of 64 ISQ buccolingually and 74 ISQ mesiodistally at placement, increasing to 76 ISQ buccolingually and 82 ISQ mesiodistally pre‐prosthetically.

**Conclusions:**

The findings suggest that immediate dental implants placed without osteotomies in select anatomical conditions can achieve successful osseointegration and functional stability. However, given the small sample size, further studies with larger cohorts are necessary to validate these results.

## Summary

- Immediate implants can achieve primary stability without osteotomy in select cases.
- Anatomical healing abutments may enhance tissue volume preservation and emergence profile.
- Further studies with larger samples are required to validate these preliminary findings.

## 1 Introduction

Traditional implant placement requires osteotomy preparation through a series of progressively increasing diameters of drills. The rationale for this was initially based on preparing osteotomies in flat osseous architecture to accommodate implant fixtures without increasing the temperature while at the same time accommodating unobstructed placement of the desired fixture (Brånemark et al. 1977). This traditional approach is challenged in scenarios of immediate implant placement in sockets when utilizing modern fixture designs with innovative macro‐geometry. This case series evaluates the feasibility of placing implants without osteotomy in fresh extraction sockets using a novel implant design. This manuscript presents 8 patients with 10 extractions and immediate implants using a novel implant fixture that predictably allows for such an approach.

## 2 Materials and Methods

### 2.1 Study Design and Participants

Eight patients requiring extraction due to non‐restorable teeth were included. A comprehensive dental and medical history review confirmed implant candidacy. Institutional Review Board (IRB) approval was not obtained, as this study is a review of clinical cases showcasing proof‐of‐concept. Informed consent was acquired from all patients. A thorough dental and periodontal exam took place in conjunction with full mouth radiography as well as preoperative cone beam computed tomography (CBCT). Informed consent was acquired, and risks, benefits, and alternatives were discussed in detail.

### 2.2 Surgical Procedure

Local anesthesia using 4% Articaine with 1,100k epinephrin took place, and hopeless teeth were removed in toto. All osseous walls were present without any dehiscence or fenestrations, and removal of any granulomatous tissue took place in conjunction with thorough socket irrigation with sterile lactated Ringer's solution. Without the use of any drills, dental implant fixtures (Megagen Anyridge, Megagen America) were immediately placed in fresh extraction sockets in ideal prosthetic orientation, verifying positioning with a vacuform matrix. Every effort was made to avoid contact of the implant fixture with the coronal portion of the buccal/facial plate. All implants were noted to have 1–2 mm gap between the fixture and the buccal/facial socket wall. Resonance frequency analysis (RFA) immediately took place post placement (W&H Osstell Beacon), and insertion torque was recorded with a calibrated implant motor (W&H Implantmed). Gaps between socket walls and fixtures were filled with cortical cancellous allograft (Mineross) and anatomical healing abutments created with the VPI Cervico system were placed (Innovato Holdings Ltd.).

### 2.3 Healing and Prosthetic Protocol

**Figure 1:**
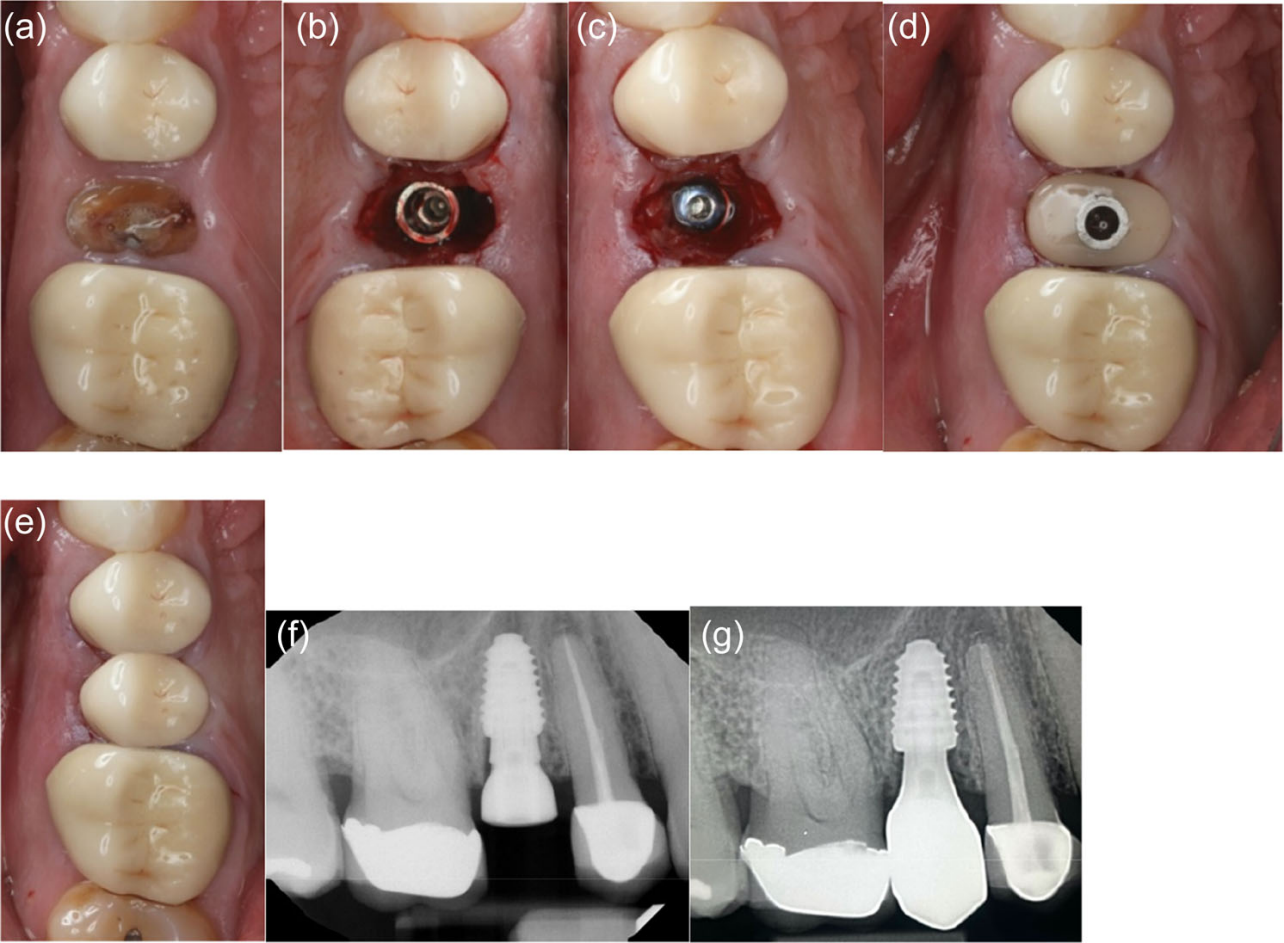
Minimally invasive removal of fractured premolar and placement of immediate implant without osteotomy on Patient 3: (a) Fractured non‐restorable premolar. (b) Prepless immediate implant. (c) Cover screw placed to accommodate grafting with cortical cancellous allograft. (d) Anatomical healing abutment placed on the day of surgery. (e) Final monolithic Zr restoration. (f) Peri‐apical radiograph of day of surgery with anatomical healing abutment. (g) Peri‐apical radiograph with definitive restoration at 12 months.

**Figure 2:**
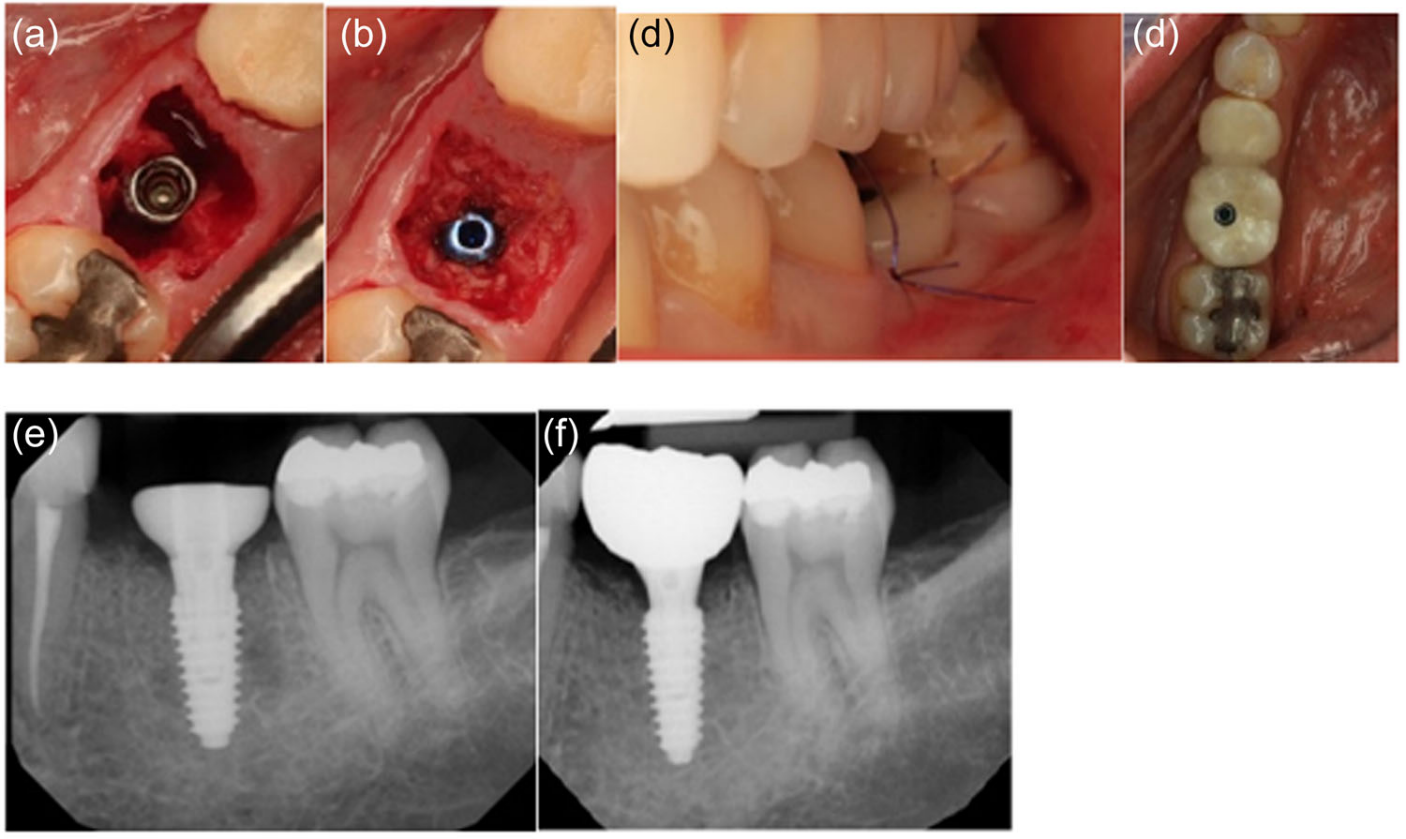
Minimally invasive removal of carious molar and placement of immediate implant without osteotomy on Patient 7. (a) Flapless and prepless Implant placement in molar socket. (b) Cover screw placed to accommodate grafting with cortical cancellous allograft. (c) Anatomical healing abutment placed to cover the entire socket and accommodate volume preservation. Sutures placed to re‐approximate papillae. (d) Final screw‐retained crown. (e) Peri‐apical radiograph on the day of placement. (f) Peri‐apical radiograph with final restoration at 12 months.

**Figure 3:**
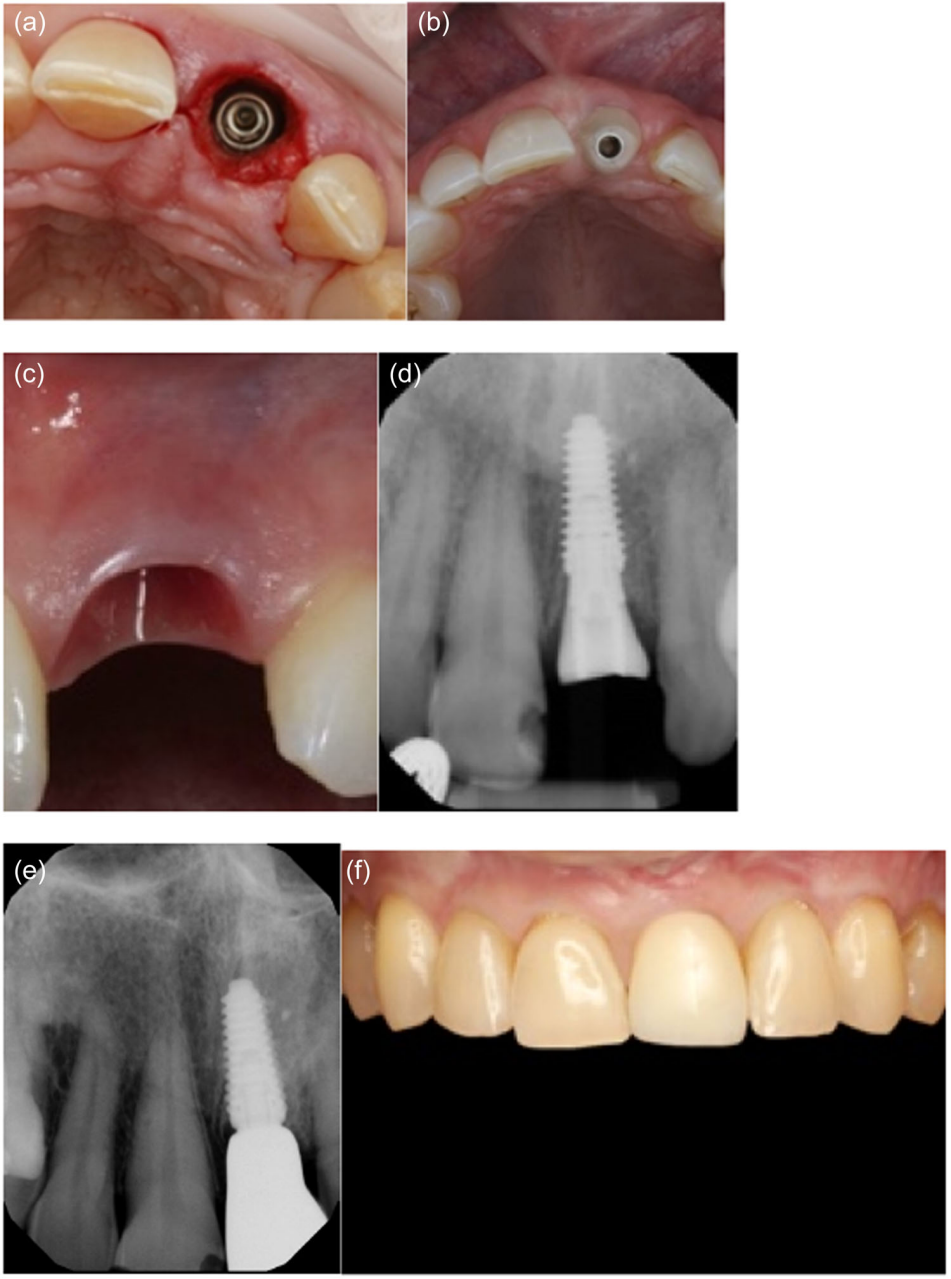
Minimally invasive removal of fractured central incisor and placement of immediate implant without osteotomy on Patient 1: (a) Immediate prepless immediate implant on central incisor. (b) Anatomical healing abutment placed. (c) Transmucosal tunnel and emergence profile development by anatomical healing abutment. (d) Peri‐apical radiograph on the day of implant placement with anatomic healing abutment. (e) Peri‐apical radiograph with final restoration at 12 months. (f) Definitive monolithic Zr restoration.

**Figure 4:**
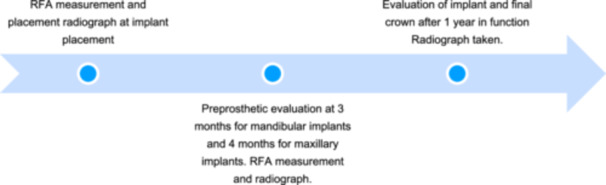
Timing of data collection.

Anatomical healing abutments were used to support the emergence profile of future restorations. In all cases, implants were allowed to osseointegrate for 4 months without any further intervention. A pre‐prosthetic evaluation took place at 4 months postoperatively, and RFA values were recorded. Also, calibrated peri‐apical radiographs were also acquired. Intraoral radiographs were performed and evaluated in each patient at (a) the time of implant placement, (b) at pre‐prosthetic evaluation 4 months post‐placement, and (c) after delivery of final restoration (Figures 1, 2, 3). A digital film holder and individual bite blocks were used to ensure reproducible parallel radiographic images. In addition, the images were obtained in the way that non‐distorted implant/abutment interface and implant threads would be clearly visible, ensuring that the radiographic image is parallel. Radiographs at the time of placement, pre‐prosthetic and post‐prosthetic radiological evaluation, and measurements were performed using the Dexis software measurement program with a magnification (20×) by one examiner who was not familiar with the study (V.J.O.). For calibration purposes, a peri‐apical radiograph was taken of an implant of known dimensions outside the mouth with the beam placed exactly perpendicular to it. Bone levels were calculated as the distance between the bone crest at the highest peri‐implant interproximal peaks and the implant abutment junction. The data collection timing is illustrated in Figure 4. This case series has been reported in line with the PROCESS Guideline (Agha et al. 2020).

## 3 Results

**Figure 5:**
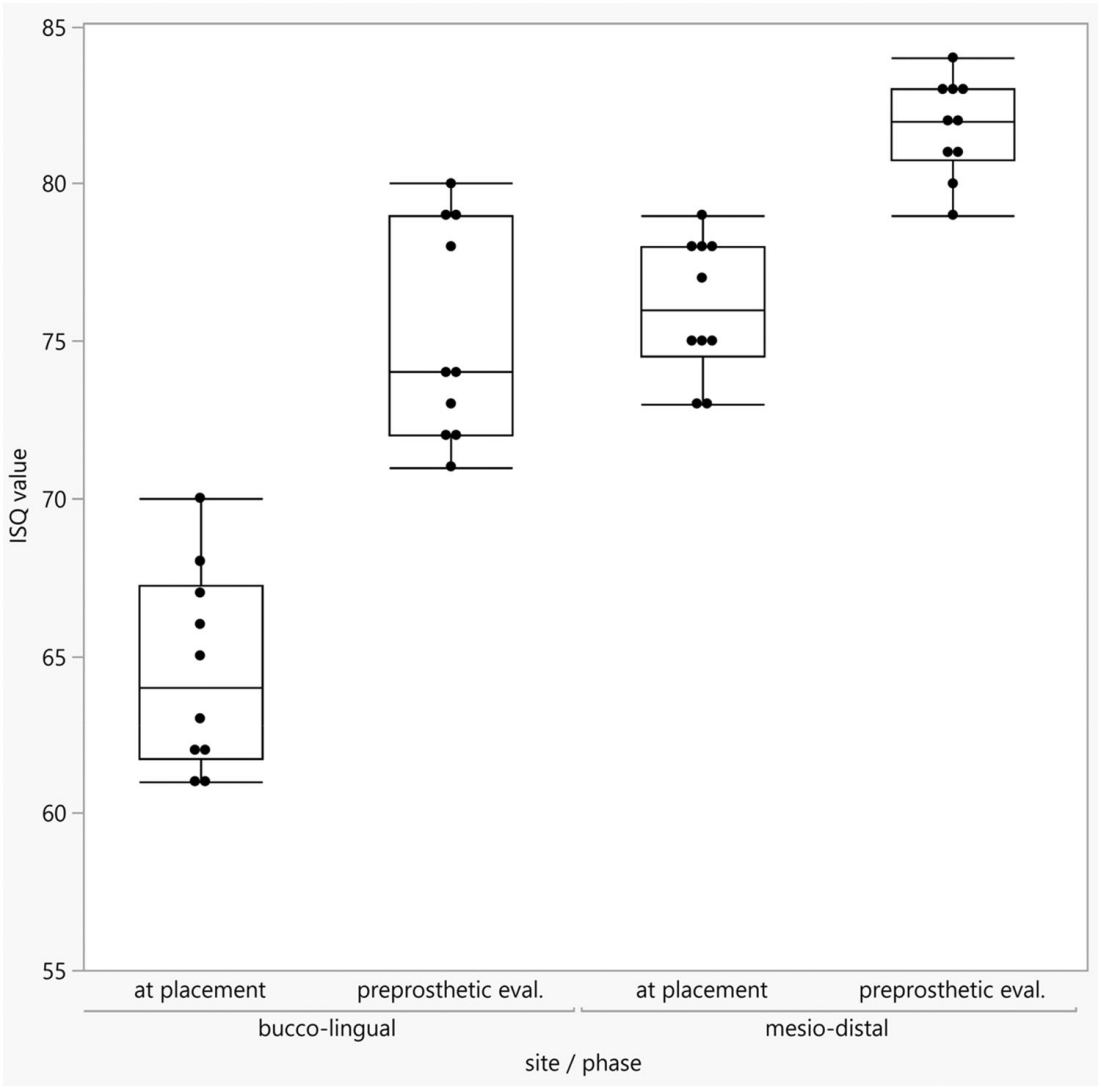
Increase in ISQ values between the two time points at implant level.

**Figure 6:**
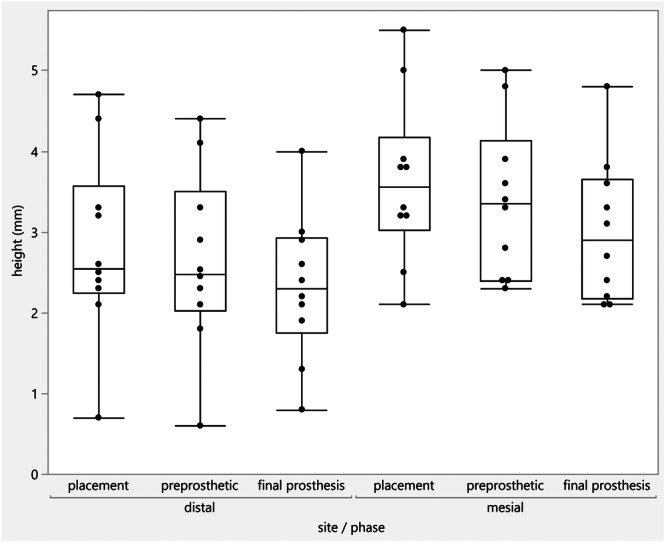
Radiographic crestal bone remodeling at placement, pre‐prosthetic evaluation, and after definitive restoration.

The patient population consisted of 8 patients: 6 females and 2 males with a median age of 66.5 years. Table 1 presents demographic data, sites of implant placement, and diameter and length of implants placed. 6 out of 10 implants placed were on premolar sites. The median insertion torque at placement was 42.5 Ncm. At 1 year follow‐up, all implants were successfully integrated and remained in function. RFA revealed a median value of 64 ISQ buccolingually and 74 ISQ mesiodistally, while the pre‐prosthetic evaluation values were 76 ISQ buccolingually and 82 ISQ mesiodistally. Figure 5 shows the increase in ISQ values between the two time points at the implant level. The median crestal bone remodeling from placement to final crowns was 0.35 mm. Crestal bone remodeling is shown in Figure 6.

**Table 1:** Demographic information of the patient sample.

Patient	Gender	Age	Site	Length	Diameter
1	F	66	#9	13	4.5
2	F	67	#13	11.5	4
3	M	90	#11	13	4.5
3	M	90	#4	10	4
4	F	58	#13	13	4
5	F	65	#28	8	4
6	M	67	#29	10	4
7	F	29	#15	10	5
7	F	29	#19	10	5
8	F	56	#13	10	4

## 4 Discussion

The macrogeometry of the used implant (Megagen Anyridge) consists of a tapered design with a narrow apex and self‐tapping variable threads with a single core diameter. This design allows for high primary stability with minimal site preparation. In addition, the internal connection is a 5‐degree Morse taper that offers reduced micro‐motion, which is known to cause bacterial infiltration and crestal bone remodeling. A tight conical connection is more critical in immediate implant placement scenarios in sockets with high scallops where the implant is placed deep interproximally (Liu and Wang 2017; Block 2022). Platform switching has been shown to allow for horizontal development of the biologic width, preserving crestal bone levels. Most bone‐level implant systems today are designed to take advantage of this feature. Studies show that an implant‐abutment mismatch of 0.4 mm may provide a more favorable bone remodeling response (Lazzara and Porter 2006; Atieh et al. 2010; Stafford 2012; Annibali et al. 2012). It is important to recognize that several other factors are critical to the overall bone remodeling, such as the peri‐implant soft tissue thickness as well as the definitive prosthetic material used (Linkevicius et al. 2009; Linkevicius and Vaitelis 2015). The combination of platform switching and a stable conical connection allows for minimal crestal bone remodeling (Block 2022). Another aspect of the technique followed in these cases is the utilization of anatomical healing abutment (AHA). AHAs have been shown to promote bone regeneration and ridge volume preservation (Corrado et al. 2023). They seal the socket, providing wound stability while preserving the dimensions of the sockets by imitating the extracted tooth dimensions at the CEJ level (Corrado et al. 2023). Utilization of AHAs allows less inflammatory infiltrate, a more oblique peri‐implant fiber orientation as well as increased soft tissue thickness (López‐López et al. 2016). In addition, the development of emergence profile by AHAs may lead to less food impaction around the definitive implant crowns in the future (Chopra et al. 2019; Chanthasan et al. 2022). The median reported bone remodeling of 0.35 mm in this limited sample, is comparable to other studies that report bone remodeling 12 months post‐implant placement (Younes et al. 2016; Palaska et al. 2016). RFA is a noninvasive method of assessing mechanical implant stability introduced by Meredith in 1996 (Meredith et al. 1996). It translates the response of a piezo‐ceramic peg to a vibratory signal into implant stability (ISQ) units. ISQ values do not directly correlate with insertion torque and are further influenced by a variety of factors such as bone quality, implant macro and micro design, bone to implant contact, and others. The literature, unfortunately, offers conflicting data on the impact of these factors on ISQ values (Huang et al. 2020). The interpretation of RFA data in assessing dental implant stability is a subject of ongoing debate within the field, primarily due to conflicting findings and a lack of clear consensus on the factors influencing RFA measurements (Chen 2019). Studies have reported inconsistent results regarding the correlation between RFA measurements, marginal bone loss, and other clinical parameters. Some investigations have shown significant associations between RFA and bone loss, while others have found no such relationship (Monje et al. 2019). The variability in study designs, patient populations, implant types, and measurement protocols makes it difficult to compare results across different studies (Huang et al. 2020). This heterogeneity further complicates the interpretation of RFA data. ISQ values are influenced by a complex interplay of factors including bone quality, implant macro and micro design, and the extent of bone‐to‐implant contact. The relative contribution of each of these factors to ISQ values is not fully understood, leading to variability in results (Chen 2019). RFA should be used as a supplementary tool in combination with radiographic assessments and other clinical examinations rather than as a standalone method of assessing implant stability. Although guidelines (Osstell, Gothenburg, Sweden), exist for interpreting ISQ values (e.g., > 70 ISQ is considered high stability), the clinical significance of these specific values is still debated. Furthermore, the trend of ISQ values over time, particularly from baseline to loading, is considered more important than any single ISQ measurement. Our study showed relatively high initial ISQ values at the time of placement that increased by the time of pre‐prosthetic evaluation when the decision to proceed with loading was made.

### 4.1 Study Limitations and Considerations

- Limited Sample Size: The small number of cases prevents broad generalization of results.
- Heterogeneous Prosthetic Restorations: Implants were restored by different general dentists without standardized prosthetic materials or lab protocols.
- Lack of Control Group: A comparative study is needed to validate outcomes against conventional osteotomy‐based implant placement.
- As reported in the literature, there are standardized methods of connecting the RFA transducers on the implants (Naughton et al. 2023). In this case series, the RFA transducers were hand tightened by the same operator (TD), according to manufacturer's instructions. This may have introduced to some variability on the RFA measurements that cannot be defined. As literature points out, it is debatable whether or not standardization of tightening forces is necessary.

### 4.2 Clinical Implications

- Implant Design: The tapered, knife‐shaped macro‐geometry facilitates primary stability without site preparation.
- Platform Switching: This approach minimizes crestal bone remodeling, aligning with literature findings.
- Anatomical Healing Abutments (AHA): These abutments optimize emergence profile, reduce inflammatory infiltrate, and promote tissue preservation.

The findings align with prior studies on RFA and bone remodeling, though some conflicting data exist on ISQ value interpretation. Future studies should include a larger cohort with standardized prosthetic protocols to refine clinical recommendations.

## 5 Conclusions

Immediate implants placed without osteotomy in fresh extraction sockets demonstrated favorable stability and minimal bone remodeling over 12 months. While promising, these findings are limited by the small sample size. Further research is needed to establish definitive clinical guidelines for this technique.

## Author Contributions

**Thanos Dounis:** conceptualization and clinical application of this study. **Valeria Jimenez Ortiz:** data collection and radiographic measurements.

## Conflicts of Interest

The authors declare no conflicts of interest.

## Supporting information

IMG 2833.

X‐ray for paper.

## Data Availability

The data that support the findings of this study are available on request from the corresponding author. The data are not publicly available due to privacy or ethical restrictions.
