# Supplementary figures and images for: Dental Implants Placed in Fresh Human Extraction Sockets Without Osteotomy: A Case Series
Source: Clin Exp Dent Res. 2025 Jun 11;11(3):e70159. doi: 10.1002/cre2.70159 (PMC12152763; doi:10.1002/cre2.70159)

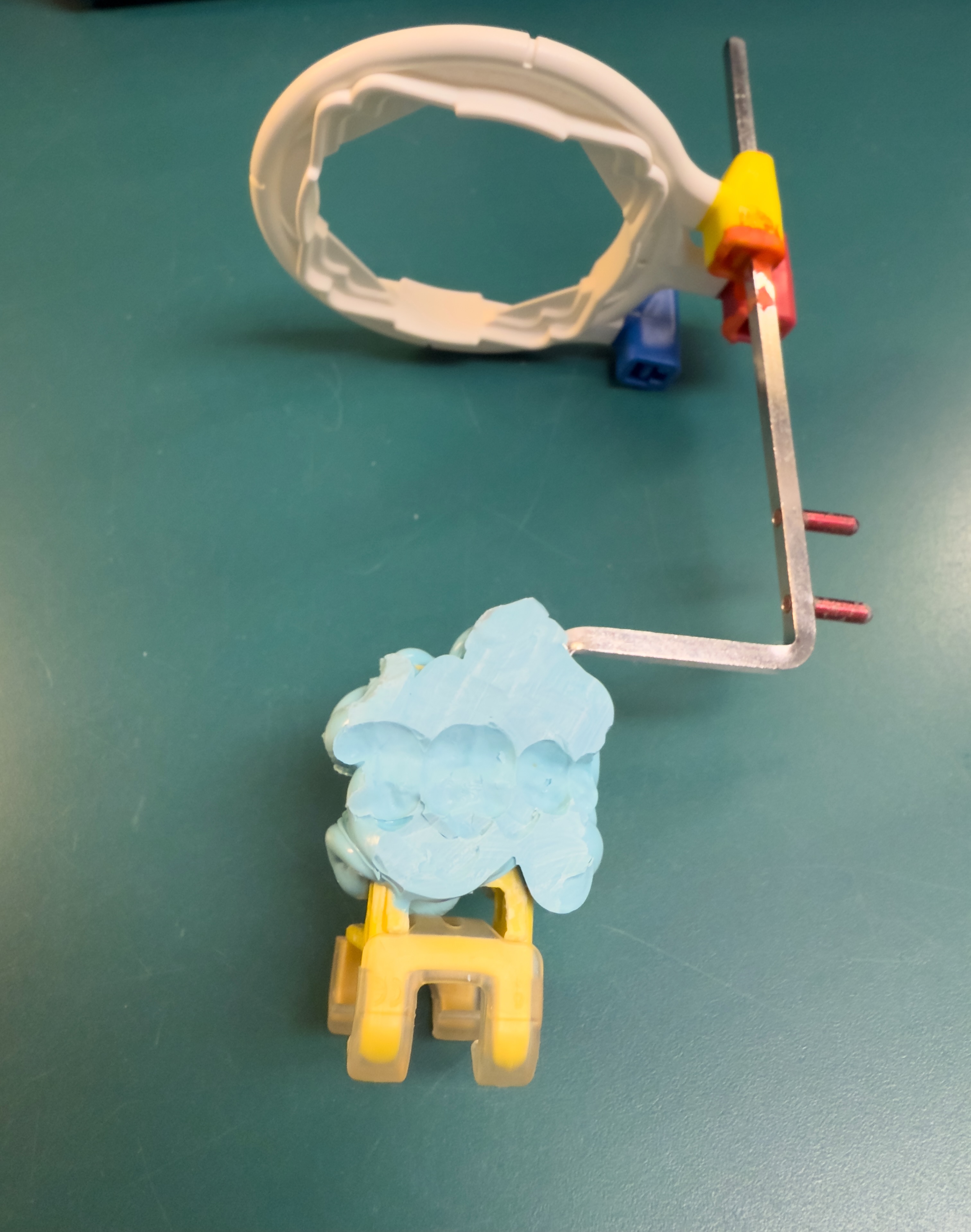

Supplement: Supplementary file 1 — IMG 2833. [file CRE2-11-e70159-s002.jpeg]

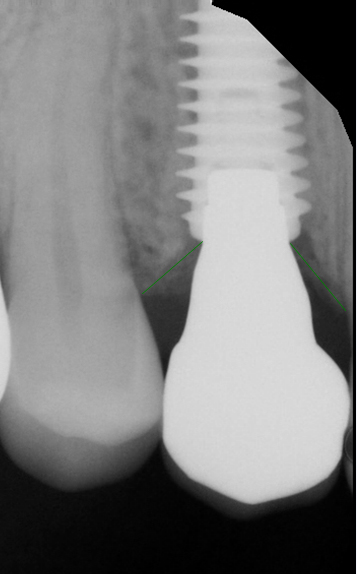

Supplement: Supplementary file 2 — X‐ray for paper. [file CRE2-11-e70159-s001.jpg]
